# Concurrent Aerobic and Strength Training with Caloric Restriction Reduces Insulin Resistance in Obese Premenopausal Women: A Randomized Controlled Trial

**DOI:** 10.3390/medicina59071193

**Published:** 2023-06-24

**Authors:** Yasser M. Aneis, Ghada E. El Refaye, Mona Mohamed Taha, Monira I. Aldhahi, Hany F. Elsisi

**Affiliations:** 1 Department of Basic Sciences, Faculty of Physical Therapy, Cairo University, Giza 11432, Egypt; 2 Department of Basic Sciences, Faculty of Physical Therapy, Delta University for Science and Technology, Gamasa 11152, Egypt; 3 Department of Physical Therapy for Women’s Health, Faculty of Physical Therapy, Cairo University, Giza 11432, Egypt; 4 Department of Physical Therapy for Women’s Health, Faculty of Physical Therapy, Pharos University, Alexandria 21311, Egypt; 5 Department of Rehabilitation Sciences, College of Health and Rehabilitation Sciences, Princess Nourah Bint Abdulrahman University, P.O. Box 84428, Riyadh 11671, Saudi Arabia; mialdhahi@pnu.edu.sa; 6 Department of Physical Therapy for Cardiovascular/Respiratory Disorders and Geriatrics, Faculty of Physical Therapy, Cairo University, Giza 11432, Egypt; 7 Department of Respiratory Therapy, College of Applied Medical Sciences, University of Bisha, Bisha 61922, Saudi Arabia

**Keywords:** aerobic exercise, caloric restriction, insulin resistance, premenopause, strength training

## Abstract

*Background and Objectives*: Obese premenopausal women are at high risk of developing insulin resistance (IR). Concurrent aerobic and strength training (CAST) has been shown to provide remarkable advantages, yet its effects, along with caloric restriction in such a high-risk population, are not yet established. This study aimed to investigate the impact of concurrent aerobic and strength training with caloric restriction (CAST-CR) on IR in obese premenopausal women. *Materials and Methods*: Forty-two obese premenopausal women with reported IR, aged 40–50 years, were randomly allocated to either the (CAST-CR) intervention group, who underwent CAST with caloric restriction, or the (AT-CR) control group, who received aerobic training in addition to caloric restriction. Both groups completed 12 weeks of controlled training with equivalent training time. Aerobic training began at 60% and gradually progressed to achieve 75% of the maximum heart rate, while strength training was executed at 50% to 70% of the one-repetition maximum (1RM). Anthropometric measures, abdominal adiposity, metabolic parameters, and homeostasis model assessment-estimated insulin resistance (HOMA-IR) were evaluated prior to and following the intervention. *Results*: Both groups experienced a substantial enhancement in the selected parameters compared to the baseline (*p* < 0.001), with higher improvement within the CAST-CR group. The changes in HOMA-IR were −1.24 (95%CI, −1.37 to −1.12) in the CAST-CR group vs. −1.07 (95%CI, −1.19 to −0.94) in the AT-CR group. *Conclusions*: While AT-CR improved insulin sensitivity in premenopausal women who were obese and hyperinsulinemic, CAST with calorie restriction improved insulin sensitivity more significantly, suggesting it as a preferable alternative.

## 1. Introduction

Insulin resistance (IR), one of the most prevalent metabolic disorders related to obesity, is linked to the development of chronic conditions such as non-insulin-dependent diabetes and cardiovascular disease. This has given rise to an extensive health problem affecting many age groups, particularly menopausal women [1].

The progression of menopause is characterized by the onset or worsening of specific risk factors associated with this phase, such as abdominal obesity, hypertension, and dyslipidemia. Such potential factors, along with hyperglycemia, hyperinsulinemia, and IR, constitute metabolic syndrome (MS) [2].

Longitudinal studies that tracked premenopausal women and compared the levels of visceral adiposity between those who underwent menopause and those who did not have shown significant increases in visceral adiposity and a higher prevalence of metabolic syndrome among postmenopausal women at the time of the study [3,4]. However, analysis of the data suggests that premenopausal women who were still being tracked had comparable or greater progress on these measures, in contrast to those who had experienced menopause [3,4,5]. The rate of change in metabolic syndrome or insulin sensitivity elements could be slow, even in women who did not use hormone replacement therapy after menopause [4,5]. The rapid progression of metabolic syndrome severity during the years leading up to menopause, rather than after menopause, is believed to be the cause of the higher prevalence of metabolic syndrome [6].

Given the high incidence of IR and its associated health burden among premenopausal women, the urgent need to establish compelling preventive strategies while improving optimal health and quality of life is highlighted. Weight reduction through caloric reduction is acknowledged to diminish and even reverse IR [7,8] and adding aerobic exercise training to a calorie-restricted diet has been proposed to boost the enhancement in insulin, and it has been suggested that improving insulin sensitivity can be achieved by combining a calorie-restricted diet with aerobic exercise training [9,10]. Alternatively, resistance training may induce advantageous changes in insulin sensitivity by means of muscle bulk enhancement, adequate expansion of glucose storage, promotion of blood glucose clearance, and a reduction in the amount of insulin required to maintain the typical glucose tolerance [11]. However, concurrent training programs that incorporate the two modalities have been approved as the most efficacious as they combine various action mechanisms [12,13,14]. To date, the therapeutic potential of concurrent aerobic and strength training (CAST) with caloric restriction on insulin sensitivity has not been established. Therefore, this study aimed to examine the impact of CAST with caloric restriction on IR in obese premenopausal women. We hypothesized that women randomly assigned to the CAST group with a caloric restriction would achieve more noteworthy improvements in insulin sensitivity.

## 2. Materials and Methods

### 2.1. Participants

**Figure 1:**
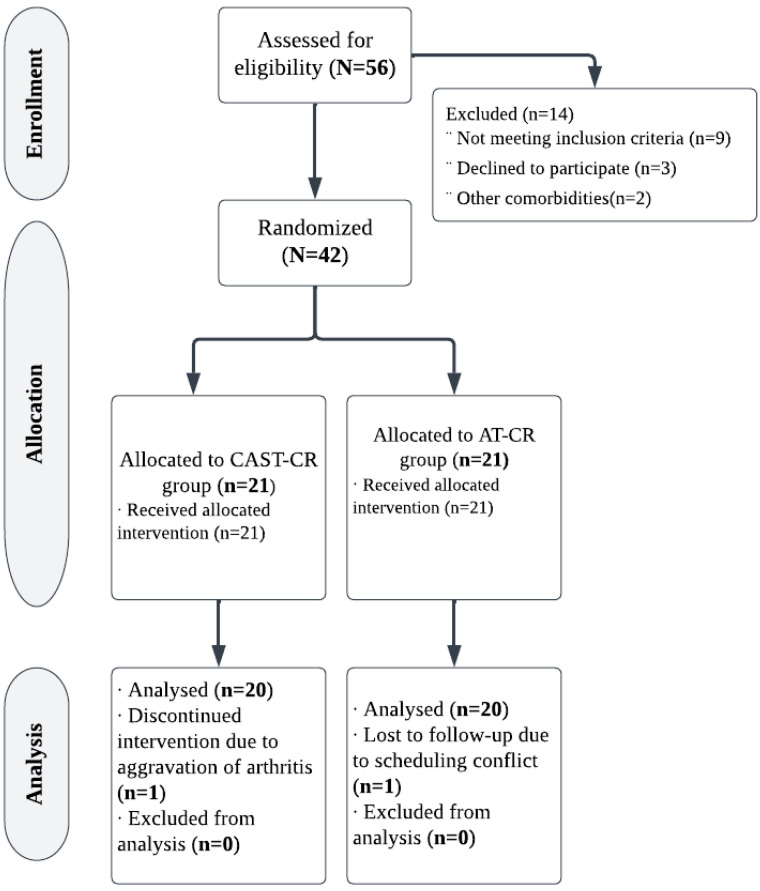
Study flowchart.

In this 12-week randomized controlled trial, forty-two obese premenopausal women (aged 40 to 50 years) were screened and divided into two groups at random: the intervention group (CAST-CR), who underwent CAST with caloric restriction, or the control group (AT-CR), who received aerobic training in addition to caloric restriction, as shown in Figure 1. They were recruited from Kasr El Aini Teaching Hospital’s outpatient clinic at Cairo University.

Patients were chosen to participate in this study upon meeting the following eligibility criteria: premenopausal women who were obese and reported insulin resistance with a HOMA-IR cutoff value of 1.62 [15], a BMI > 30, a waist circumference more than 88 cm, and a stable weight for six months prior to the study’s start. They also had to live a sedentary lifestyle (no engagement in an organized workout throughout the preceding year), and not take any medications, including contraceptive pills, that were thought to influence the key outcome measures. The menopausal status was reported through the assessment of follicle-stimulating hormone levels (less than 30 IU/L).

Subjects with coronary artery dysfunction, diabetes mellitus, cerebrovascular and peripheral vascular disorders, high blood pressure, hypothyroidism, and persistent bronchial disease, as well as those on drugs known to have an effect on physical activity or metabolism or those who were unable to perform the essential testing procedures, were excluded. Table 1 includes the participant’s demographic and clinical features.

### 2.2. Randomization

To avoid bias, random assignments of the participants were carried out in two phases. First, fellow physiotherapists working in ambulatory clinics identified all women who satisfied the study’s inclusion criteria and did not have a criterion for exclusion. Second, women were assigned randomly in a 1:1 ratio to either the CAST-CR or AT-CR groups following a medical consultation through the launching of an obscure envelope designed by an unbiased subject with random number creation. Patients had given informed legal consent to participate in the study and to generalize the results. This work was officially approved by the Institutional Review Board of the Faculty of Physiotherapy, Cairo University (no. P.T.REC/012/002156), registered in the Clinical Trials database: Clinicaltrials.gov NCT03716336, and is in conformity with CONSORT guidelines. All of the women were given a full explanation of the study’s methodology and goals, and were instructed to maintain their usual everyday activities and lifestyle throughout the study.

### 2.3. Evaluative Procedures

#### 2.3.1. Anthropometric Parameters

At baseline and after 3 months, anthropometric measures were taken while participants wore hospital gowns without shoes. Height was evaluated with a standard scale on a portable stadiometer to the closest 0.1 cm with bare feet, and a standard weight scale was used to measure body weight to the nearest 0.1 kg while the subject was wearing light clothing. At the end of a typical expiration, the waist circumference was assessed utilizing a constant tension tape (inelastic tape), with arms loose along the sides, at the midpoint between the lowest part of the lower rib and the highest point of the hip in the mid-axillary axis. The BMI was calculated by dividing a person’s weight in kilograms by their height in meters squared.

#### 2.3.2. Abdominal Adiposity

Visceral adipose tissue (VAT) and subcutaneous fat were assessed using magnetic resonance imaging with a 1.5 Tesla magnet (Siemens Magnetom Symphony, Erlangen, Germany). Using a gradient echo “in phase” and “out phase” sequence, a single slice at the L4 level was used to evaluate adipose tissue distribution. The classification system of Shen et al. [16] was used to define visceral adipose tissue. Fat that is enclosed by the visceral cavity is referred to as visceral fat. The VAT compartment encompassed adipose tissue located within the abdominal muscle walls, including preperitoneal, intraperitoneal, and retroperitoneal adipose tissue [17]. The subcutaneous fat thickness (SFT) was evaluated by measuring the supraumbilical diameter of subcutaneous fat in the axial slice [18].

#### 2.3.3. Blood Sampling and Analysis

Venous blood samples were taken from an antecubital vein at approximately 8:00 a.m. by a qualified technician after an overnight fast. Low-density lipoprotein (LDL), total cholesterol, high-density lipoprotein (HDL), triglyceride, hepatic enzymes, alanine aminotransferase (ALT), and aspartate aminotransferase (AST) were measured by a standard kit (CELM, Barueri, Brazil). Concentrations of blood glucose were estimated through a glucose oxidase technique (Glucose-Analyzer II, Beckman Industries, Fullerton, USA). An insulin enzyme-linked immune-sorbent assay kit (ALPCO, Salem, NH, USA) was used to assess plasma insulin levels. Homeostasis model assessment-estimated insulin resistance (HOMA-IR) was utilized to estimate IR. Fasting blood glucose (FBG) and immunoreactive insulin (I) were used to calculate HOMA-IR as follows: (HOMA-IR = fasting insulin (μIU/mL) × fasting glucose (mmol/mL)/22.5.) [19].

#### 2.3.4. One-Repetition Maximum

One-repetition maximum (1RM) is the highest amount of load that can be borne while effectively finishing one repetition of an exercise. Initially, the test begins with a warm-up of 6 to 10 repeats with approximately half of the expected load for the primary attempt in the 1RM test. Following two minutes of rest, patients were directed to try to achieve two repetitions. If two repetitions were accomplished in the first experiment, or even if neither was accomplished, a second repetition was undertaken with a workload higher or lower than that given in the previous attempt after a three-to-five-minute recovery period. These approaches were further refined in a third and final attempt in the event that a single repetition maximum was not yet established [20].

### 2.4. Intervention

#### 2.4.1. Exercise Training Protocols

Both exercise groups underwent a 12-week supervised training program with a similar amount of training time, working out for 60 min per day, three days per week. Sufficient warming up and cooling down in the form of large muscle stretching, flexibility exercises, breathing exercises, and low-intensity gait (50% of maximum heart rate (max HR)) were attempted for all subjects. Adequate time was given to familiarize patients in the resistive group by enabling them to execute one set of each workout on various weight machines. Similarly, enough time was given to familiarize patients with the treadmill and safety concerns within the aerobic training group.

#### 2.4.2. Aerobic Exercise Training Protocol

Following a warm-up, on nonconsecutive days, patients in this group achieved walking using a treadmill at a modest speed without inclination three times a week. The intensity of exercise was specified according to the Karvonen equation, while the target heart rate = [(max HR − resting HR) × % intensity] + resting HR, where max HR = 220-age. Patients began training with 60% of max HR and gradually progressed to achieve 75% of max HR by weeks 7 through 12 [20]. During all exercise sessions, a heart rate monitor (Polar Electro, Kempele, Finland) was equipped to control the appropriate workload. The exercise training protocol is illustrated in (Table A1, Appendix A).

#### 2.4.3. Concurrent Exercise Training Protocol

CAST was executed three times a week over 12 weeks, consisting of 30 min of resistance training and 30 min of aerobic exercise (participants adopted the same previously described aerobic training but just for 30 min). Resistance training was conducted prior to aerobic training to prevent premature fatigue that may result from aerobic exercise [21]. The resistance training incorporated seated rows, bench presses, biceps curls, triceps pushdowns, leg presses, leg curls, calf raises, squats, abdominal crunches, and lower back extensions. During the first six weeks, the exercises were executed with 50–60% of 1RM in 2 sets of 10 repetitions and a recovery time of 90–120 s. However, the intensity of the workout rose in the subsequent weeks to 65–70% of 1RM in 3 sets of 8 repetitions [20]. All subjects were asked to strictly adhere to the sequence of exercises and to avoid engaging in any other organized physical activity during the intervention. The exercise training protocol is illustrated in (Table A2, Appendix B)

#### 2.4.4. Dietary Intervention

During the baseline period, participants engaged in an individualized nutritional session where the study dietitian provided proper food preparation and selection. Throughout this session, daily energy needs were defined by estimating resting energy expenditure. Subjects were directed to diminish their caloric intake by 500–1000 kcal a day, with the aim of achieving a weight reduction of 10% through a low-calorie diet (LCD). A diet with a daily caloric intake of between 800 and 1200 kcal is referred to as an LCD. The LCD was composed of 30% fat, 15% protein, 55% carbohydrates, and 20–30 g of fiber/day [22]. Individual adherence to the recommended dietary plan was tracked from self-reported food logs, and changes in body weight were collected and reviewed weekly.

### 2.5. Outcome Parameters

The primary outcome was the change in HOMA-IR. Changes in anthropometric measures, abdominal adiposity, and metabolic parameters were secondary outcomes. Assessments were carried out at the beginning and following 12 weeks of intervention.

### 2.6. Blinding

Double blinding was used, where patients and assessors who performed all assessments were unaware of the patients’ treatment group.

### 2.7. Statistical Analysis

The normality and homogeneity of variance were checked before performing the final analysis using the Shapiro–Wilk and Levene tests, and no violations were found for any of the dependent variables. The adherence of all participants to the diet program was estimated, where χ^2^ examined the degree of adherence to the recommended dietary plan. To estimate the main effect between and within groups, a two-way multivariate mixed model analysis of variance (MANOVA) was used. When a significant time–group interaction effect was found, additional univariate ANOVAs (two-way mixed models) were conducted. Bivariate correlations between the variables of interest were evaluated through Pearson correlation coefficients. Multiple linear regression analyses were performed to identify the independent predictors of HOMA-IR changes. The independent variables in these analyses were chosen based on their associations with the dependent variable in bivariate analyses. Statistical analyses were performed using SPSS version 25.0 (SPSS, Inc., Chicago, IL, USA). The level of significance was set at a *p*-value < 0.05.

## 3. Results

G*Power (version 3.0.10, Germany) was used to calculate the sample size. With two independent groups, a sample size of 34 patients was considered sufficient based on F tests (MANOVA: effects and interactions) with Type I error = 0.05, power (1-error probability) = 0.80, and effect size = 0.50. To account for the possibility of dropout, 40 patients were recruited (assuming a 15% dropout rate). The progress of participants from recruitment to follow-up is illustrated in Figure 1. Of the 56 women evaluated for eligibility, 42 (75%) were allocated randomly to groups. Two (4.7%) of the forty-two eligible patients missed follow-up. Consequently, 20 women in each group adhered to the intervention and achieved follow-up. 

The data analysis revealed no significant differences in patients’ demographic and anthropometric characteristics between the two groups prior to intervention (*p* > 0.05), as shown in Table 1. No side effects attributable to the intervention were recorded among patients.

Repeated measures MANOVA revealed a significant main effect of time (Wilks’ Λ = 0.01, F(13, 26) = 186.68, *p* = 0.0001, η^2^ = 0.98), treatment (Wilks’ Λ = 0.36, F(13, 26) = 3.45, *p* = 0.003, η^2^ = 0.63), as well as a significant time–treatment interaction (Wilks’ Λ = 0.28, F(13, 26) = 5.05, *p* = 0.0001, η^2^ = 0.71). Follow-up univariate ANOVAs indicated a considerable change in BMI, F(1, 38) = 61.55, *p* < 0.001, η^2^ = 0.61; WC, F(1, 38) = 338.69, *p* < 0.001, η^2^ = 0.89; total cholesterol, F(1, 38) = 282.69, *p* < 0.001, η^2^ = 0.88; HDL cholesterol, F(1, 38) = 124.23, *p* < 0.001, η^2^ = 0.76; LDL cholesterol, F(1, 38) = 634.84, *p* < 0.001, η^2^ = 0.94; triglycerides, F(1, 38) = 252.49, *p* < 0.001, η^2^ = 0.86; AST, F(1, 38) = 228.81, *p* < 0.001, η^2^ = 0.85; ALT, F(1, 38) = 405.50, *p* < 0.001, η^2^ = 0.91; SFT, F(1, 38) = 108.54, *p* < 0.001, η^2^ = 0.74; VAT, F(1, 38) = 108.35, *p* < 0.001, η^2^ = 0.74; fasting glucose, F(1, 38) = 90.01, *p* < 0.001, η^2^ = 0.70; fasting insulin, F(1, 38) = 876.54, *p* < 0.001, η^2^ = 0.95; and HOMA-IR, F(1, 38) = 711.99, *p* < 0.001, η^2^ = 0.94. This indicates that there are differences between groups in a linear combination of outcomes between the pre- and post-intervention periods.

Following the intervention, the two groups had substantial reductions in BMI, WC, total cholesterol, LDL cholesterol, triglycerides, AST, ALT, SFT, VAT, fasting glucose, fasting insulin, and HOMA-IR, as well as an increase in HDL cholesterol (*p* < 0.001).

Between-group analyses revealed a significant difference between the two groups, with the CAST-CR group exhibiting the most significant alterations, where the mean differences at a 95% confidence interval were (−3.15, −1.80) for BMI, (−9.13, −5.96) for WC, (−31.28, −25.65) for total cholesterol, (15.32, 8.45) for HDL cholesterol, (−27.58, −18.69) for LDL cholesterol, (−41.62, −36.72) for triglycerides, (−16.50, −12.80) for AST, (−9.65, −7.29) for ALT, (−1.67, −1.07) for SFT, (−2092.59, −1316.25) for VAT, (−0.79, −0.46) for fasting glucose, (−6.43, −5.47) for fasting insulin, and (−1.24, −1.07) for HOMA-IR (Table 2 and Table 3).

Multiple regression analyses revealed that the change in HOMA-IR after treatment was best predicted by BMI (β-coefficient = 0.40, *p* < 0.0001), LDL cholesterol (β-coefficient = 0.51, *p* = 0.004), triglycerides (β-coefficient = 0.68, *p* < 0.0001), ALT (β-coefficient = 0.28, *p* = 0.03), VAT (β-coefficient = 0.35, *p* < 0.001), fasting insulin (β-coefficient = 0.51, *p* < 0.0001), and HOMA-IR at baseline (β-coefficient = 0.386, *p* = 0.014), Table 4.

The subjects’ diet adherence in the CAST-CR and AT-CR groups was 90% and 85%, respectively. Diet adherence had no significant relationship with groups (*p* = 0.68), as shown in Table 5. Additionally, there was no difference between groups in terms of daily dietary total energy intake.

## 4. Discussion

The transition to menopause is associated with adipose tissue storage changes that lead to android body composition, increasing the risk of cardiovascular disease and type 2 diabetes in postmenopausal women [23]. A limited number of studies have examined the impact of exercise and weight reduction on insulin resistance in women during the transition to menopause. Mandrup et al. stated that high-intensity exercise training for three months resulted in a similar reduction in both subcutaneous fat and visceral adipose tissue mass in either pre- or postmenopausal women [23]. A cross-sectional study examined the impact of habitual physical activity on anthropometric measures and cardiovascular risk factors in premenopausal, perimenopausal, and postmenopausal women. The study found that middle-aged women who walked 6000 or more steps per day had a lower risk of cardiovascular disease and diabetes, regardless of their menopause status [24]. Lin et al. found [25] that men, premenopausal women, and postmenopausal women experienced similar weight loss (5–6%) and body composition changes with short-term alternate-day fasting. The study also found that key metabolic risk factors such as insulin resistance, fasting insulin, and blood pressure improved similarly in all three groups. Although the impact of exercise and weight loss on insulin resistance appears to be consistent across different stages of menopause, early detection and management of insulin resistance are crucial for preventing long-term health complications.

In this study, the effects of CAST with calorie restriction on HOMA-IR, anthropometric measures, abdominal adiposity, and metabolic parameters in obese premenopausal women were compared to those of the control group who received aerobic exercise in addition to caloric restriction. Following 12 weeks of intervention compared to baseline, both groups exhibited a significant enhancement in the selected parameters, with the CAST-CR group showing higher improvement. Between-group differences were noteworthy (*p* < 0.05).

The improvement in HOMA-IR observed following exercise and calorie restriction may be attributed to the changes in anthropometric measures, abdominal adiposity, and metabolic markers. In multiple regression analyses, HOMA-IR at baseline and changes in BMI, LDL cholesterol, triglycerides, ALT, VAT, and fasting insulin were the best predictors of HOMA-IR improvement.

Exercise in women is related to a remarkable increment in lipolysis of abdominal adipose tissue relative to femoral adipose tissue [26], suggesting that exercise-driven weight reduction will be concomitant with preferentially diminished abdominal obesity. Our findings revealed that CAST-CR was more effective for anthropometric parameters relative to AT with caloric restriction. These come in line with results from both pairwise and network meta-analyses indicating that CAST is the most successful way of enhancing anthropometric markers of adiposity [27,28,29].

Our findings revealed a significant reduction in both visceral adiposity and subcutaneous fat post-intervention, with the CAST-CR exhibiting the most significant changes. These results are consistent with previous reports demonstrating that weight loss using exercise and a low-calorie diet reduces subcutaneous and intra-abdominal fat in obese women [30,31], and that the CAST reduces abdominal obesity more effectively than aerobic exercise alone [31,32,33].

Cross-sectional studies have shown that abdominal obesity is a strong predictor of the development of IR [34,35]. However, our results demonstrated that the reduction in visceral adiposity is a key element in the modulation of IR. Subclinical chronic inflammation is one of the possible processes behind visceral fat’s role in IR development [36].

Abdominal adipose tissue can be regarded as an endocrine organ because of its ability to release adipokines and different substances which are intimately related to IR [37]. The progression of IR is significantly influenced by oxidative stress and low-grade chronic inflammation, and inflammatory cytokines are hypothesized to have a role in the relationship between inflammation, oxidative stress, and IR [38]. Unfavorable changes in adipokines and inflammatory markers are closely associated with expanded visceral adiposity at menopause [39].

It has been proven that AT and dietary interventions are effective strategies for regulating inflammatory processes from visceral elements, including a rise in adiponectin and a decrease in adult inflammatory markers [40]. Nonetheless, concurrent exercise has been shown to be more effective than AT-only protocols in increasing adiponectin concentrations, indicating a significant impact on regulating the inflammatory processes associated with obesity [41].

Weight reduction through lowering fat mass decreases the systemic free-fatty acid content in the bloodstream, consequently diminishing its accessibility to skeletal muscle tissue [42]. This will restrain the aggregation of intramuscular lipids as well as other fatty acid derivatives that are known to impair insulin signaling in the insulin-resistant muscle, where basal fat oxidation is compromised [43]. Adding exercise to calorie-restricted diet regimens improves mitochondrial consistency and skeletal muscle capillarization [44] and initiates enzymes engaged in fatty acid transport and oxidation [45]. The metabolic rate of an individual is considerably influenced by the mitochondrial proton leak in skeletal muscles. This leak is facilitated by carrier proteins located on the inner membrane of the mitochondria, including adenine nucleotide translocator (ANT) and uncoupling protein-3 (UCP3) [46,47,48,49]. The formation of higher-order supercomplexes (SCs) by complexes I, III, and IV of the mitochondrial electron transport chain (ETC) can also help to improve energy transduction efficiency and reduce reactive oxygen species (ROS) production [50]. Exercise has been found to enhance mitochondrial function in skeletal muscles, which can lead to improved body composition in women who have undergone minimal weight loss through diet [51].

Furthermore, expanded utilization of substrates [52] with diminished oxidation of carbohydrates and augmented muscle glucose transport might be taken into account [53]. Consequently, it is possible that calorie restriction combined with exercise would result in more significant improvements in substrate metabolism and insulin sensitivity [54].

The effect of aerobic training on insulin resistance has been extensively studied, and it has been found to improve muscle insulin sensitivity by increasing the number of insulin receptors [55]. This leads to an increase in insulin binding to monocytes, allowing the muscle to use glucose properly, especially during exercise, and reducing glucose levels [56]. Studies using 31P NMR spectroscopy have shown that aerobic training (AT) can increase nonoxidative glucose metabolism throughout the body by 60% to 70% and insulin sensitivity by 40% or more. This is due to an increase in glucose transport phosphorylation, which leads to greater glycogen synthesis [54]. Moreover, studies have reported increases in nonoxidative glucose disposal accompanied by increased GS mRNA and insulin-stimulated glucose uptake of 60% [57], as well as increases in muscle GLUT4, HK2, AS160, Akt1/2, and insulin-responsive aminopeptidase protein levels and activity [58]. Other factors that may contribute to the improvement in insulin resistance include changes in fiber type [59], enhanced blood flow, changes in mitochondrial oxidative capacity [57], altered muscle lipid metabolism [60], and endocrine/paracrine mediators, as well as reduced visceral obesity [61].

Furthermore, a resistive exercise involving independent, brief activation of single muscle groups leads to an increase in glucose transporter type 4 (GLUT-4) content and insulin receptors due to muscle mass outgrowth [62]. This was confirmed by Sigal et al. [63], who reported a more prominent improvement in glycemic control with combined aerobic and strength training than with either aerobic or strength training alone. 

Additionally, the restoration of insulin sensitivity through aerobic and resistance training might be attributed to the adenosine monophosphate-activated protein kinase (AMPK) pathway [64]. Biochemically, exercise-induced boosts in AMPK are thought to facilitate glucose uptake and free-fatty oxidation while limiting lipid synthesis [65]. However, training programs incorporating the two modalities could have greater potential to combat insulin resistance.

One of the key advantages of our experiment is the use of magnetic resonance imaging (MRI), a precise and widely accepted standard method for determining changes in abdominal adiposity. Additionally, we meticulously examined a number of intermediate parameters that could potentially elucidate the effect of exercise and calorie restriction on insulin resistance (IR). However, it is important to acknowledge the limitations of our trial. We evaluated insulin resistance using the Homeostatic Model Assessment of Insulin Resistance (HOMA-IR) test, which is widely used in clinical research, but the hyperinsulinemic–euglycemic clamp is considered the gold standard for assessing insulin action. Nevertheless, it is also a more invasive and expensive procedure. Furthermore, we did not investigate the long-term effects of caloric restriction with adequate protein and exercise (CAST) on IR in obese premenopausal women, which would be valuable to investigate in future studies.

## 5. Conclusions

This trial proposes evidence that significant improvements in insulin sensitivity can be achieved through 12 weeks of CAST with caloric restriction. These results suggest that this exercise regimen may be a superior option for premenopausal women with obesity and hyperinsulinemia. Consequently, such findings may provide an effective non-pharmacological therapy for healthcare professionals to alleviate IR in premenopausal women who are obese and hyperinsulinemic.

## Figures and Tables

**Table 1:** Patients’ demographic and anthropometric features at baseline.

Variables	CAST-CR Mean ± SD	AT-CR Mean ± SD	*p*-Value
Age (year)	44.72 ± 3.26	43.62 ± 3.45	0.540
Height (cm)	161.07 ± 4.79	160.37 ± 5.73	0.890
Weight (kg)	94.34 ± 8.62	96.72 ± 12.21	0.640
BMI (kg/m^2^)	38.64 ± 2.47	35.47 ± 2.55	0.410
Waist Circumference (cm)	119.53 ± 5.57	118.35 ± 4.38	0.461

CAST-CR, concurrent aerobic and strength training with caloric restriction; AT-CR, aerobic training with caloric restriction; BMI, body mass index; SD, standard deviation; level of significance at *p* < 0.05.

**Table 2:** Anthropometric measures, abdominal adiposity, and metabolic features pre- and post-intervention ^b^.

Characteristics		CAST-CR (Mean ± SD)	AT-CR (Mean ± SD)	ANOVAs (Between-Groups)
BMI (kg/m^2^)	Pre-intervention	38.64 ± 2.47	35.47 ± 2.55	0.01 *
	Post-intervention	35.49 ± 2.28 ^a^	33.66 ± 2.07 ^a^	
	MD (95% CI)	−3.15	−1.80	
Waist Circumference (cm)	Pre-intervention	119.53 ± 5.57	118.35 ± 4.38	0.22
	Post-intervention	110.39 ± 6.16 ^a^	112.38 ± 3.66 ^a^	
	MD (95% CI)	−9.13	−5.96	
Total Cholesterol (mg/dL)	Pre-intervention	219.04 ± 8.03	221.93 ± 9.88	0.318
	Post-intervention	187.75 ± 6.57 ^a^	196.27 ± 8.22 ^a^	0.001 *
	MD (95% CI)	−31.28	−25.65	
HDL Cholesterol (mg/dL)	Pre-intervention	49.32 ± 9.04	50.24 ± 6.34	0.712
	Post-intervention	64.62 ± 4.80 ^a^	58.69 ± 4.65 ^a^	0.001 *
	MD (95% CI)	15.32	8.45	
LDL Cholesterol (mg/dL)	Pre-intervention	128.29 ± 8.91	126.16 ± 7.16	0.411
	Post-intervention	100.70 ± 6.24 ^a^	107.46 ± 6.81 ^a^	0.002 *
	MD (95% CI)	−27.58	−18.69	
Triglycerides (mg/dL)	Pre-intervention	157.76 ± 20.93	164.74 ± 20.01	0.286
	Post-intervention	116.13 ± 10.68 ^a^	128.04 ± 12.54 ^a^	0.003 *
	MD (95% CI)	−41.62	−36.72	
AST (U/L)	Pre-intervention	44.85 ± 8.79	46.85 ± 6.51	0.419
	Post-intervention	28.35 ± 4.60 ^a^	34.05 ± 5.14 ^a^	0.001 *
	MD (95% CI)	−16.50	−12.80	
ALT (U/L)	Pre-intervention	38.21 ± 3.75	38.84 ± 3.43	0.582
	Post-intervention	28.56 ± 2.60 ^a^	31.55 ± 3.35 ^a^	0.003 *
	MD (95% CI)	−9.65	−7.29	
SFT (cm)	Pre-intervention	3.33 ± 1.03	3.26 ± 0.98	0.826
	Post-intervention	1.66 ± 0.54 ^a^	2.18 ± 0.99 ^a^	0.04 *
	MD (95% CI)	−1.67	−1.07	
VAT (cm^3^)	Pre-intervention	3542.35 ± 1269.02	3391.21 ± 986.57	0.676
	Post-intervention	1449.75 ± 728.27 ^a^	2074.95 ± 907.07 ^a^	0.02 *
	MD (95% CI)	−2092.59	−1316.25	
Fasting Glucose (Mmol/L)	Pre-intervention	4.74 ± 0.44	4.85 ± 0.45	0.424
	Post-intervention	3.94 ± 0.42 ^a^	4.38 ± 0.29 ^a^	0.001 *
	MD (95% CI)	−0.79	−0.46	
Fasting Insulin (uIU/mL)	Pre-intervention	10.26 ± 1.10	9.62 ± 0.96	0.092
	Post-intervention	3.83 ± 1.06 ^a^	4.15 ± 0.75 ^a^	0.276
	MD (95% CI)	−6.43	−5.47	
HOMA-IR	Pre-intervention	1.83 ± 0.23	1.88 ± 0.25	0576
	Post-intervention	0.59 ± 0.24 ^a^	0.80 ± 0.25 ^a^	0.009 *
	MD (95% CI)	−1.24	−1.07	

CAST-CR, concurrent aerobic and strength training with caloric restriction; AT-CR, aerobic training with caloric restriction; BMI, body mass index; HLD, high-density lipoprotein; LDL, low-density lipoprotein; AST, aspartate aminotransferase; ALT, alanine aminotransferase; SFT, subcutaneous fat thickness; VAT, visceral adipose tissue; HOMA-IR, homeostasis model assessment insulin resistance; MD, mean difference; SD, standard deviation; CI, confidence interval; ^a^ significant *p* value between baseline and 12 weeks within groups; ^b^ adjustment for pairwise multiple comparisons: Bonferroni; * significant; level of significance at *p* < 0.05.

**Table 3:** Between-group effects after 3 months of intervention *.

Characteristics	CAST-CR Group versus AT-CR Group	Partial Eta Squared
	MD (95% CI)	
BMI (kg/m^2^)	1.83 (0.43, 3.22) ^a^	0.26
Waist Circumference (cm)	−1.99 (−5.23, 1.25)	0.04
Total Cholesterol (mg/dL)	−8.51 (−13.28, −3.75) ^a^	0.25
HDL Cholesterol (mg/dL)	5.94 (2.92, 8.97) ^a^	0.29
LDL Cholesterol (mg/dL)	−6.76 (−10.95, −2.57) ^a^	0.22
Triglycerides (mg/dL)	−11.90 (−19.36, −4.44) ^a^	0.21
AST (U/L)	−5.70 (−8.82, −2.57) ^a^	0.26
ALT (U/L)	−2.98 (−4.90, −1.06) ^a^	0.20
SAT (cm)	−0.52 (−1.03, −0.01)	0.15
VAT (cm^3^)	−625.19 (−1151.76, −98.62) ^a^	0.23
Fasting Glucose (Mmol/L)	−0.44 (−0.67, −0.20) ^a^	0.27
Fasting Insulin (uIU/mL)	−0.32 (−0.91, 0.26)	0.03
HOMA-IR	−0.21 (−0.37, −0.05) ^a^	0.18

CAST-CR, concurrent aerobic and strength training with caloric restriction; AT-CR, aerobic training with caloric restriction; BMI, body mass index; HLD, high-density lipoprotein; LDL, low-density lipoprotein; AST, aspartate aminotransferase; ALT, alanine aminotransferase; SAT, subcutaneous adipose tissue; VAT, visceral adipose tissue; HOMA-IR, homeostasis model assessment insulin resistance; MD, mean difference; CI, confidence interval; *, Data are mean ± SD; ^a^ significant *p* value between-group effects after 3 months of intervention, level of significance at *p* < 0.05.

**Table 4:** Predictors of change in insulin sensitivity by multiple regression analysis.

Variables in the Model	β-Coefficients	95% CI	*p*-Value
Change in HOMA-IR (R^2^ = 0.804, *p* < 0.0001)			
HOMA-IR at baseline	0.386	(0.093, 0.772)	0.014
BMI (kg/m^2^)	0.404	(0.015, 0.078)	0.005
LDL Cholesterol (mg/dL)	0.514	(0.007, 0.031)	0.004
Triglycerides (mg/dL)	0.682	(0.020, 0.008)	0.0001
ALT (U/L)	0.285	(0.002, 0.044)	0.033
VAT (cm^3^)	0.351	(0.128, 0.344)	0.001
Fasting Insulin (uIU/mL)	0.511	(0.090, 0.208)	0.0001

BMI, body mass index; LDL, low-density lipoprotein; ALT, alanine aminotransferase; VAT, visceral adipose tissue; HOMA-IR, homeostasis model assessment insulin resistance; CI, confidence interval; level of significance at *p* < 0.05.

**Table 5:** Subjects’ adherence to the recommended dietary plan.

Relationship between the Groups and Degree of Adherence					
Groups		Adherence		Total	*p*-Value
		Adherent	Non-Adherent		
CAST-CR	Count	18	2	20	0.69 ^a^
	Expected count	16.3	3.7	20	
	% within groups	90%	10%	100%	
AT-CR	Count	17	3	20	
	Expected count	16.3	3.7	20	
	% within groups	85%	15%	100%	
Total	Count	35	5	40	
	Expected count	32.5	7.5	40	
	% within groups	78.5%	12.5%	100%	
Daily dietary total energy intake					
		CAST-CR (Mean ± SD)		AT-CR (Mean ± SD)	*p*-value
Daily caloric intake (kcal)		1027.3 ± 88.4		1106.3 ± 57.6	0.10 ^a^
Fat (%)		32.3 ± 6.1		32.1± 6.4	0.81 ^a^
Protein (%)		17.2 ± 6.5		17.6 ± 5.1	0.87 ^a^
Carbohydrate (%)		47.6 ± 6.2		49.3 ± 7.4	0.42 ^a^

CAST-CR, concurrent aerobic and strength training with caloric restriction; AT-CR, aerobic training with caloric restriction; % fat, protein, and carbohydrates were calculated based on the total daily energy intake; ^a^ value estimated by χ^2^; level of significance at *p* < 0.05.

## Data Availability

The corresponding author will provide the identified datasets used in the current study upon reasonable request.

## Appendix A

**Table A1:** Aerobic exercise training protocol.

Weeks	Order	Exercise	Intensity (%Max HR)	Time	Frequency
12 weeks	Warming up	Static stretching		5 min	Three times a week on non-sequential days
		Breathing exercise			
		Low-intensity gait			
	Dynamic stage	Walking on a treadmill at modest speed without inclination	1–2 weeks: 60% of max HR	60 min	
			3–4 weeks: 65% of max HR		
			5–6 weeks: 70% of max HR		
			7–12 weeks: 75% of max HR		
	Cooling down	Static stretching		5 min	
		Breathing exercise			
		Low-intensity gait			

## Appendix B

**Table A2:** Concurrent exercise training protocol.

Weeks	Order	Exercise	Intensity	Time	Volume	Frequency
12 weeks	Warming up	Static stretching Breathing exercise Low-intensity gait		5 min		Three times a week on non-sequential days
	Resistance training	Seated rows Bench press Bicep curl Triceps pushdowns Leg press Leg curls Calf raise Squat Abdominal crunch Lower back extension	1–2 weeks: 50% of 1RM 3–4 weeks: 55% of 1RM 5–6 weeks: 60% of 1RM 7–8 weeks: 65% of 1RM 9–12 weeks: 70% of 1RM	30 min	1–6 weeks: 2 sets of 10 repetitions 7–12 weeks: 3 sets of 8 repetitions	
	Aerobic training	Walking on a treadmill at moderate speed with no tendency	1–2 weeks: 60% of max HR 3–4 weeks: 65% of max HR 5–6 weeks: 70% of max HR 7–12 weeks: 75% of max HR	30 min		
	Cooling down	Static stretching Breathing exercise Low-intensity gait		5 min		
